# An Enhanced Multiple Sclerosis Disease Diagnosis via an Ensemble Approach

**DOI:** 10.3390/diagnostics12071771

**Published:** 2022-07-21

**Authors:** Hanaa Torkey, Nahla A. Belal

**Affiliations:** 1 Computer Science and Engineering Department, Faculty of Electronic Engineering, Menoufia University, Menouf 32952, Egypt; htorkey@el-eng.menofia.edu.eg; 2 College of Computing and Information Technology, Arab Academy for Science, Technology, and Maritime Transport, Smart Village 12577, Egypt

**Keywords:** ensemble learning, multiple sclerosis, diagnosis, gene expression, differentially expressed genes

## Abstract

Multiple Sclerosis (MS) is a disease attacking the central nervous system. According to MS Atlas’s most recent statistics, there are more than 2.8 million people worldwide diagnosed with MS. Recently, studies started to explore machine learning techniques to predict MS using various data. The objective of this paper is to develop an ensemble approach for diagnosis of MS using gene expression profiles, while handling the class imbalance problem associated with the data. A hierarchical ensemble approach employing voting and boosting techniques is proposed. This approach adopts a heterogeneous voting approach using two base learners, random forest and support vector machine. Experiments show that our approach outperforms state-of-the-art methods, with the highest recorded accuracy being 92.81% and 93.5% with BoostFS and DEGs for feature selection, respectively. Conclusively, the proposed approach is able to efficiently diagnose MS using the gene expression profiles that are more relevant to the disease. The approach is not merely an ensemble classifier outperforming previous work; it also identifies differentially expressed genes between normal samples and patients with multiple sclerosis using a genome-wide expression microarray. The results obtained show that the proposed approach is an efficient diagnostic tool for MS.

## 1. Introduction

Multiple Sclerosis (MS) is a chronic inflammatory disease that attacks the central nervous system. MS is characterized by lesions in the brain and spinal cord, which cause various neurological symptoms, including, but not limited to blindness, double vision, muscle weakness, and changes in sensation and balance [1]. Multiple sclerosis ranges from the relapsing form, where attacks are over long intervals, to the progressive form, where symptoms progressively get worse over time. It is classified into Clinically Isolated Syndrome (CIS), Relapsing–Remitting MS (RRMS), Primary Progressive MS (PPMS), and Secondary Progressive MS (SPMS) [2]. To the present date, there is no known cure for MS, nor the causes of the disease. Some mention the cause to be the destruction by the immune system or the failure of cells that produce myelin to protect the nerves; others mention genetics or viral infection [3].

Multiple sclerosis is known to be one of the most common auto-immune disorders. The reports about the global burden of disease [4] revealed that, in 2015, about 2.3 million people were diagnosed as MS patients, with about 18,900 deaths, as opposed to 12,000 deaths from MS in 1990.

In [5], a study involving patients with Pediatric-Onset MS (POMS) was carried out to evaluate changes in the prognosis of POMS over time with associated therapeutic changes. It was shown that the risk of persistent disability was reduced by 50% to 70% in recent diagnosis epochs. Furthermore, in [6], Late-Onset Relapsing–Remitting MS (LORRMS) and Young-Onset Relapsing–Remitting MS (YORRMS) were studied, and it was shown that the male population with LORRMS reached severe disability faster than those with YORRMS. Moreover, [7] studied more symptoms associated with MS. The study showed that severe disease course is associated with a higher risk of neuropathic pain. Furthermore, as shown in [8], dysphagia is one of the frequent MS symptoms.

This work is motivated by the fact that patients have an increased risk of being poorly diagnosed, hence increasing the risk factors and deterioration due to attacks. In addition to the availability of data and the increased diagnostic performance achieved by machine learning, machine learning has recently shown promising results for many neurological disorders. Proposed methods have successfully distinguished healthy subjects from patients with high accuracy. However, none of the proposed diagnostic methods have achieved reliable levels of accuracy to identify the genes associated with the disease as a basis for diagnosis. Moreover, none of the work in the literature has addressed the class imbalance nature of most of the MS expression data samples. Furthermore, most of these studies rely on data that are rarely used in clinical routine. In addition to that, ensemble learning has proven to be an effective approach for improving disease diagnosis accuracy.

Many researchers addressed the problem of multiple sclerosis prediction from different perspectives. However, the literature still lacks efficient predictive models that benefit from all the available data. In this section, the most cited and recent work in multiple sclerosis is summarized. Furthermore, the section briefly covers the work related to the imbalance dataset problem in diseases. Some work in the literature addressed the association of certain genes with the disease. Random forests were used in [9] to identify new genes associated with MS.

Weygandt et al. [10] used SVM to classify Relapsing–Remitting MS (RRMS) patients. The highest accuracy reported on 44 patients and 26 healthy controls was 95% using brain lesions, given that lesions are much more frequent in MS patients than in healthy controls, and also, normal-appearing tissue can be affected by microstructural changes [11].

Bendefeldt et al., in [12], employed Support Vector Machines (SVMs) to perform binary classification in MS to classify patients with a short disease duration (less than 5 years) and a long disease duration (more than 10 years), low T2 lesion load (less than 1 mL) and high T2 lesion load (more than 10 mL), and benign MS (with an Expanded Disability Status Scale (EDSS) less than or equal to 3) and non-benign MS (EDSS more than 3). The accuracy reported was 85%, 83%, and 77%, respectively.

Chen et al. [13] proposed the Voxelwise Displacement Classifier (VDC); a classifier based on Fisher’s linear discriminant analysis, SVM, Random Forest(RF)and Adaboost when using displacement fields as features. The study was tested on 29 Relapse–Remitting MS (RRMS), 8 Secondary Progressive MS (SPMS), 4 CIS, and 1 Primary Progressive MS (PPMS) patients and 36 healthy controls. The proposed VD classifier consistently outperformed other methods, reaching up to 100% accuracy.

Other methods used gene expression data for diagnosing multiple sclerosis. The study [14] proposed a classification model for gene selection using gene expression data, The method proposed was applied on a total of 44 samples, 26 multiple sclerosis patients and 18 individuals with other neurological diseases (control). An accuracy of 86% was achieved using an analytical framework integrating feature ranking algorithms and a support vector machine model for selecting genes associated with multiple sclerosis.

Sweeney et al. [15] compared a supervised machine learning techniques using different feature vectors extracted from MRI images to predict MS. The research concluded that the choice of the feature vector has a higher impact on the predictive performance than the machine learning algorithm used.

A method for the diagnosis of multiple sclerosis using combined clinical data with lesion loads and magnetic resonance metabolic features was presented in [16]. Three classifiers were used in the study, Linear Discriminant Analysis (LDA), Support Vector Machines (SVMs), and Random Forest (RF). The results reported in the paper suggest that metabolic features obtain good results to discriminate between relapsing–remitting and primary progressive forms, while lesion loads are better at discriminating between relapsing–remitting and secondary progressive forms. Therefore, combining clinical data with magnetic resonance lesion loads and metabolic features can improve the discrimination between relapsing–remitting and progressive forms.

Margineau et al. [17] used four binary classifiers to classify multiple sclerosis courses using features extracted from Magnetic Resonance Spectroscopic Imaging (MRSI) combined with brain tissue segmentations of gray matter, white matter, and lesions. Values of the area under the curve ranged between 68% and 95%, with the highest percentage recorded for Support Vector Machines with a Gaussian kernel (SVM-rbf) applied on MRSI features combined with brain tissue segmentation features. According to their comparison, their work concluded that combining metabolic ratios with brain tissue segmentation percentages obtained high classification results for Clinically Isolated Syndrome (CIS), Relapsing–Remitting (RR), and Primary Progressive (PP) patients. The best results were obtained with SVM-rbf, and therefore, building complex architectures of convolutional neural networks does not add any improvement over classical machine learning methods.

Ostemeyer et al. [18] used statistical learning to diagnose immune diseases. Their method was a repertoire-based statistical classifier for diagnosing Relapsing–Remitting Multiple Sclerosis (RRMS) with an accuracy up to 87%. Moreover, this method points to a diagnostic biochemical motif in the antibodies of RRMS patients. Zhao et al. [19] used SVMs and compared them to logistic regression on 1693 CLIMB patients using demographic, clinical, and MRI data. The study showed that SVMs improved predictions and outperformed linear regression in most cases.

In [20], machine learning techniques were employed on microarray datasets to identify defective pathways related to MS. The analysis resulted in a list of highly discriminatory genes, where the most discriminatory genes were related to the production of Hemoglobin. The analysis also revealed coincidences of MS with some viruses, such as Epstein–Barr virus, Influenza A, Toxoplasmosis, Tuberculosis, and Staphylococcus Aureus infections.

MS could also be diagnosed with some biomarkers as shown in the work in [21]. The biomarkers suggested in [21] are the plasma levels of Tumor Necrosis Factor (TNF)-α, soluble TNF Receptor (sTNFR) 1, sTNFR2, adiponectin, hydroperoxides, Advanced Oxidation Protein Products (AOPPs), nitric oxide metabolites, total plasma antioxidant capacity using the Total Radical-trapping Antioxidant Parameter (TRAP), Sulfhydryl (SH) groups, and serum levels of zinc. Support vector machines were used on 174 MS patients and 182 controls and achieved a training accuracy of 92.9% and a validation accuracy of 90.6%. The results showed that MS is characterized by lower levels of zinc, adiponectin, TRAP, and SH groups and higher levels of AOPPs.

In 2020, Zhao et al. [22] extended the work performed in 2017 in [19] and used 724 patients from the Comprehensive Longitudinal Investigation in MS at Brigham and Women’s Hospital (CLIMB study) and 400 patients from the EPIC dataset, University of California, San Francisco, to continue the study on MS prediction. This paper used SVM, logistic regression, and random forest, in addition to the ensemble learning approaches XGBoost, LightGBM, and Meta-learner L. The research concluded that ensemble methods give higher predictive performance of MS disease.

In [23], support vector machines were used to diagnose MS using the plasma levels of selenium, vitamin B12, and vitamin D3. The study used 99 MS patients and 81 healthy controls. The supervised machine learning methods used were the support vector machine algorithm, decision tree, and K-nearest-neighbor. The highest accuracy, 98.89%, was achieved using support vector machines.

Shang et al. [24] used gene expression data to uncover genes associated with MS. the authors performed bioinformatics analysis to identify differentially expressed genes and also explored the potential SNPs associated with MS. The study provided identified genes, SNPs, biological processes, and cellular pathways associated with MS.

Another important aspect considered in this paper is imbalanced data. A dataset is considered to be imbalanced when the number of samples representing one class is significantly fewer than the other classes. The class with the fewest number of samples is called the minority class, and the others are the majority classes. Handling the class imbalance data problem is of great interest, as shows in many real-world problems, especially medical diagnosis and disease prediction. Training classifiers on these datasets makes the classifiers more biased towards the majority classes, as the rules that predict the majority class samples are positively weighted, whereas the rules that predict the minority class are usually treated as noise or ignored. This leads to the conclusion that the minority class samples are prone to misclassification [25]. Class-imbalance-aware methods either modify the standard classifiers used or incorporate a data-driven approach in the training process to deal with the different class sample sizes. Under-sampling and over-sampling are the data-driven techniques most commonly used for handling class imbalance datasets. Recently, much research has been presented to handle this problem. For instance, Garcia et. al. [26] presented a data-level ensemble approach for class imbalance data based on GACE meta-heuristics and feature space adaptive partitioning. From the results, this system was able to reduce the time complexity and improve the imbalanced classification accuracy. An approach presented by K. Pasupa et. al. [27] modified the stander classification process for the Convolutions Neural Network (CNN). A focal loss function for a classification task was employed to handle the data imbalance problem in a CNN deep learning model. Comparing the focal loss function with the cross-entropy function proved that the focal loss function can enable the deep learning model to be less biased towards the class with majority samples.

One of the flaws of these algorithm-based methods is that they require specific modifications to the classification algorithm, which makes it lack generality and a systematic approach for evaluating the performance of different classifiers. Classification methods are developed to predict the class of the future samples based on the assumption that the training samples are good examples of the future samples, where matching the frequency of classes in training data would warrant a realistic performance. Moreover, data-driven down-sampling methods lead to missing some of the majority class samples, which could have significant information for the classifier.

It is concluded from the background and related work discussed in the Introduction above that the diagnosis of MS is an extensible process with variable possible types of data that can be used, as well as several techniques. In the proposed model, we relied on gene expression data to diagnose MS based on the fact that gene expression identifies a unique group of genes in a cell that occurs as a result of an altered or unaltered biological process or pathogenic medical condition. As most of the expression profile datasets available for MS are imbalanced, our proposed model adapts an over-sampling technique to handle class imbalance expression data.

In this paper, a hierarchical ensemble approach that employs voting and boosting ensemble techniques is proposed. Ensemble methods are used to improve the predictive classification performance of the constituent classifiers by using multiple learning algorithms [28]. Two ensemble techniques are used, voting, where several base models are trained and the decision is made based on the votes of each estimator, and boosting, which is mainly used to reduce bias and variance in order to make the estimators stronger. The proposed method adapts a heterogeneous voting approach using two base learners, random forest and support vector machine. The classification probabilities of the these base learners are combined to develop a voting ensemble technique based on the majority vote of class probability to obtain the final accuracy for the ensemble approach. In training each base learner, a boost approach is employed to improve the learners’ accuracy and reduce the false prediction rate. To the best of our knowledge, very few prior studies in gene expression analysis have tried to employ an over-sampling technique to handle the class imbalance problem. Therefore, in our approach’s boosting step, each learner is trained in a balanced subset from the samples, selected from the original majority class and the over-sampling produced for the minority class. The proposed approach performance is evaluated and compared with the base learner and one of the most recently published ensemble algorithms on five KEEL imbalanced datasets, as well as the MS expression data [29]. For MS diagnosis, the experiments are carried out to evaluate the methods’ performance with all feature sets, differentially expressed genes, and reduced feature subsets from three Feature Selection (FS) algorithms. The FS algorithms employed are the Chi-squared algorithm [30], as one of the most used algorithms for feature selection, Recursive Feature Elimination with Support Vector Clustering (RFE-SVC), as a gold standard wrapper algorithm that outweighs other FS algorithms [31], the Extreme Gradient Boosting (XGBoost) for feature selection (BoostFS) [32], and DEGs generated from the linear model of the Limma package [33]. Limma is an R package developed for microarray data differential expression analysis. Moreover, Gene Ontology and KEGG Pathway enrichment analysis are performed for the identified DEGs, using the EnrichR 2.0 package [34] to reveal their functional relation to MS pathways. Accuracy, the Matthew Correlation Coefficient (MCC), Root-Mean-Squared Error (RMSE), F-score, Receiver Operating Characteristic (ROC) curve, and Area Under the Curve (AUC) are used as the evaluation metrics. Experimental results show that the proposed method achieves the highest accuracy of 92.81% and 91.8% with BoostFS and DEGs, respectively.

The contributions of this paper are summarized in the following points:

- Proposing a hierarchical ensemble approach to improve the classification accuracy for multiple sclerosis patients.
- Handling the class imbalance problem using over-sampling to improve the classifier performance when predicting the minority class.
- Applying three different feature selection algorithms, in addition to selecting genes using bioinformatics analysis on differentially expressed genes, to select relevant genes based on their importance to overcome the low number of samples compared to the number of features (microarray genes set).
- Identifying the differentially expressed genes between normal and multiple sclerosis patients’ expression profiles to use as one of the selected feature sets for evaluating our proposed approach.
- Evaluating the proposed ensemble approach to analyze the predictive accuracy on each selected feature set from the FS algorithms and DEGs. The evaluation metrics calculated are the accuracy, MCC, RMSE, F-score, AUC, and ROC curve.

The remainder of this paper is organized as follows. Section 2 presents the proposed framework. Following the methods, all experiments, the dataset, and results are given in the Results Section 3. Finally, a discussion, conclusions, and future directions are given in Section 4.

## 2. Materials and Methods

In this section, the methods and data used to conduct the study are discussed.

### 2.1. Multiple Sclerosis Data Description

The GEO database (https://www.ncbi.nlm.nih.gov/geo (accessed on 26 April 2022)) provides open access to many gene expression microarray datasets. The gene expression profile from GSE41850 [35], GSE24427 [36], GSE19285 [37], and GSE13732 [38] raw and series matric data was downloaded and analyzed in this paper. The platform for this study was established using Affymetrix Human Exon 1.0 ST Array. The GSE41850 dataset contains 816 whole-blood transcription profiling samples for 195 MS patients and 66 normal individuals. MS patient samples were taken at three stages of the disease: at the discovery stage (baseline), after one year of follow-up, and after two years of follow-up. The control samples were taken from the healthy individuals at two time points. The GSE24427, GSE19285, and GSE13732 microarrays were sampled in the same fashion as the first dataset. In order to combine the features/genes from the two datasets, the gene IDs from the two datasets were converted to the gene-symbol, and the genes that were common in both datasets were extracted to obtain 18,122 shared genes as our study features set. Table 1 shows the classes in the two datasets used for evaluating MS classification using the proposed approach. In the performance evaluation of the proposed method, the dataset was divided into two segments, 80% for training and 20% for testing.

**Figure 1:**
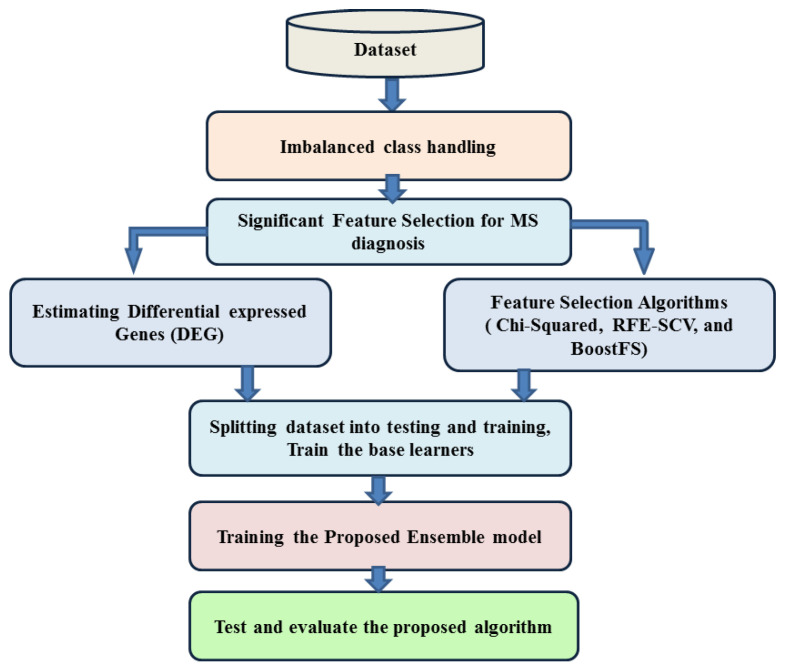
Proposed model framework.

In Figure 1, the proposed framework is shown. The framework starts by a data preprocessing step in order to deal with the missing features and normalize the gene expression values; this is explained in the Data Preprocessing Section. Following preprocessing, we estimate the over-samplings, and then, the feature selection algorithms are used to select significant feature attributes. Three feature selection algorithms were used, as illustrated in the Feature Selection Section, namely Chi-squared, Recursive Feature Elimination using Support Vector Clustering (RFE-SVC), and feature importance with the XGBoost ensemble algorithm (BoostFS). Moreover, bioinformatics analysis on differentially expressed genes was used as a method for feature selection. Finally, the classification is explained in the Classification Section. An ensemble learning approach is proposed for MS diagnosis to improve the accuracy of prediction. The suggested ensemble approach uses Random Forest (RF) and Support Vector Machine (SVM) as the base learner. The proposed approach uses both voting and boosting techniques, with RF and SVM for the voting step and LighBoost in the boosting step. The reason for using RF, SVM, and LighBoost will be justified in the Base Learner Subsection.

### 2.2. Data Preprocessing

Preprocessing is a necessary stage for ML algorithms to account for missing values (NAN) and to standardize the data range by normalization so that all features would have the same value range (between 0 and 1). Missing data could be handled in many ways, among which are weighted K-nearest-neighbor, expectation maximization, or local-least-squares approaches. In this study, missing data were replaced by the mean value of their represented class, as many studies proved that using the mean or median to replace missing values in gene expression datasets performs as well as other more complex strategies [39].

#### Imbalanced Class Data Handling

In most of the existing methods, under-sampling techniques are often used with gene expression datasets to balance the data. Under-sampling leads to missing important data; hence, the proposed approach adopts an over-sampling, specifically selective over-sampling based on the SPIDER algorithm presented by J. Stefanowski and S. Wilk [40]. The new samples are created using the k-nearest-neighbors (KNNs) of the minority class samples. KNN is a similarity-based algorithm in which the nearest neighbors are used to determine the class of each instance by identifying the group for the closest neighbors. KNN uses the Euclidean distance to estimate the sum of the absolute differences among the opposite samples in vectors *x* and *y*, where $x=(x_{1},x_{2},\cdots ,x_{n})$ and $y=(y_{1},y_{2},\cdots ,y_{n})$, as shown in Equation (1).

(1) $$D\left(x,y\right)=\sqrt{\sum_{i=1}^{n}|x_{i}-y_{i}{|}^{2}}$$

The algorithm consists of two steps. The first step is recognizing the instances of the minority and majority classes as misclassified by KNN, then labeling these samples as noisy and excluding them or their nearest neighbors from generating the new samples. In the second step, the rest of the minority class samples are divided into weak and strong samples. Then, the new samples are generated by selecting from their nearest neighbors. The selection strategy differs for the weak and strong samples. Algorithm 1 shows the detailed algorithm for handling the class imbalance problem. Using this technique for over-sampling can ensure solving the class imbalance problem in gene expression datasets, by generating new samples through random interpolation of the minority and majority samples, which can avoid the overfitting problem. Algorithm 1 uses the following functions:

- Perfect (D, s, k)—returns true or false based on the classification result of a sample *s* using its k-nearest-neighbors in set *D* for correct and incorrect classification.
- Label (D, c, f)—returns a subset of samples from set *D*∈ class *c* and labeled as *f*.
- Knn (D, s, k, c, f)—identifies the samples among the k-nearest-neighbors of *s*∈ set *D*∈ class *c* and labeled as *f*.
- Increase (D, x, k, c, f)—adds to sample *s* by finding its knn (D, s, k, c, f) and adding this subset to set *D*.

**Algorithm 1:** Imbalanced class data handling.

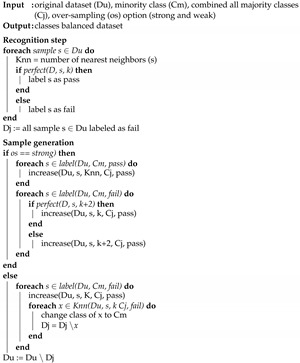



### 2.3. Feature Selection

In this section, the estimation of the differentially expressed genes is outlined, as well as the three methods used for feature selection, namely Chi-squared, RFE-SVC, and feature importance with the XGBoost ensemble algorithm (BoostFS).

#### 2.3.1. Estimating Differentially Expressed Genes

In order to obtain the DEGs, we first downloaded the raw data for the GSE41850 [35] and GSE24427 [36] raw and series metric data. The datasets were combined, and preprocessing was preformed. Preprocessing included background correction, probe summarization, and normalization, using the R package PreProcess (http://oompa.r-forge.r-project.org (accessed on 26 April 2022)). Following the preprocessing step, the limma Bioconductor package [33] was used to map the probes to genes. There are cases where multiple probes of the same genes are merged by their mean values. The linear model for the Limma package was used to discover the DEGs. The MS patient dataset was divided into three subsets to conduct the differential expression analysis. These subsets were baseline (at the first diagnosis of the disease), the first-year follow-up, and the second-year follow-up. Then, the analysis was performed between the samples of different subsets.

#### 2.3.2. Chi-Squared

The Chi-squared test [30] is a non-parametric hypothetical test for counting data with a wide range. It mainly compares between two or more sample rates and estimates the correlation analysis of the two-variable category. In feature selection, the Chi-squared test estimates stats between each feature (genes) and the classes (MS patient or normal). Then, a score is used to select features with the highest values for the test from the dataset. This algorithm results in excluding the features that are likely to be irrelevant for the classes.

#### 2.3.3. Recursive Feature Elimination using SVC

RFE-SVC is a non-linear kernel algorithm used to rank features, and ranking results could be interpreted in terms of the degree of association between the features, their association with the target (classes: patient or normal), as well as the association magnitude. In this study, Sanz et al.’s [31] estimation of the position of the features of a classification problem was used. Although RFE-SVC is time consuming, the time decreases as the number of iterations is scaled down, and it outperforms most of the existing RFE-based algorithms.

#### 2.3.4. Feature Importance with Ensemble Algorithm (BoostFS)

EXtreme Gradient Boosting (XGBoost) is one of the dominant machine learning techniques. It implements gradient boosted decision trees designed to speed up and enhance the classification performance. In the training process of XGBoost, the importance score of each feature is calculated in order to improve the efficiency of trees’ generation [41]. In this study, the XGBoost algorithm was used to classify all features by estimating the Feature Importance (FI) and sorting them in descending order. Next, the algorithm filters the features with their FI score using different cut-off values. The selected features are finally fed to the classifier to obtain the most significant feature set for MS classification.

### 2.4. Classification

In this paper, we propose an ensemble learning approach that combines two main techniques, voting and boosting. The voting technique is based on using multiple models of different learners to obtain predictions for each data point. The predictions by each model are considered for voting, and the final prediction is chosen based on the majority of the models, the average of predictions from all models, or the weighted average predictions.

**Figure 2:**
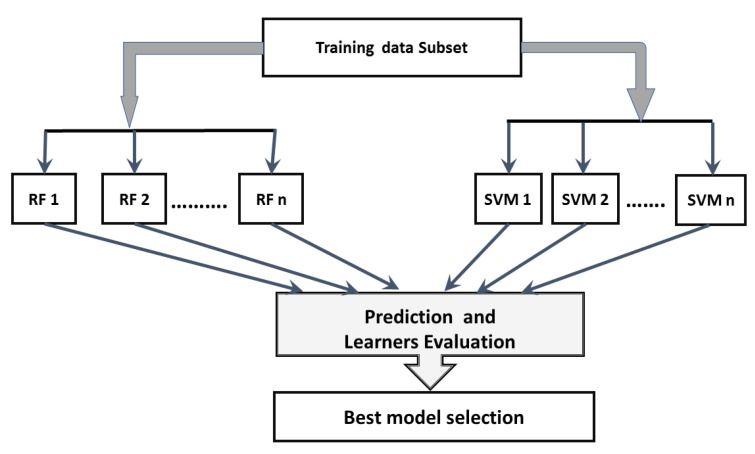
Proposed method in voting stage.

On the other hand, boosting is a sequential process on the data, where each subsequent model attempts to correct the errors of the previous model. In the boosting technique, the weighting of the samples changes over time to allow the technique to optimize its decision by considering the results from the samples in proportion to their (positive) impact on overall system accuracy. Initially, the samples are equally weighted, and after each iteration, the samples that are correctly assigned are weighted lower than the incorrectly assigned samples. The proposed approach merges the idea of voting and boosting. Random Forest (RF) and Support Vector Machine (SVM) are used as the base learners for the voting models in each iteration of the voting step. Figure 2 represents the voting process in our model.

In each voting step, the boosting technique is applied on each learner. The boosting starts by selecting a subset from the original dataset. All data points are assigned equal weights. Then, two base learners are trained on the subsets and used to make predictions on the whole dataset. The prediction errors are estimated, and the data points that are incorrectly predicted are given higher weights. The widely used boosting approaches are XGBoost [32], LightBoost [42], and AdaBoost [43]. In the implementation of the proposed approach, LightBoost was employed due to its faster training speed with large datasets, lower memory usage, and higher prediction accuracy compared to other boosting algorithms [44].

#### The Base Learner Algorithm

In the proposed ensemble approach, two base classifiers were employed, namely random forest and support vector machine. The implementation details and parameters optimization for these techniques are explained below.

The **Random Forest (RF)** algorithm is an extension of decision trees [45]. Decision trees work by constructing a tree structure that works on a set of conditions. Each internal node carries a test attribute, and the branches have conclusions for this test. Every leaf node represents a class label. The major challenge of decision trees is choosing an attribute for the nodes at every level. The best known metrics used for selecting attributes are the Gini index and information gain [46]. A weak point of decision trees is their poor accuracy; this is due to the fact that they fragment datasets and prefer majority classes. To overcome this problem, RF builds a forest of decision trees on the samples, then obtains the prediction from each tree and selects the most-voted result as the final prediction. Each individual tree takes its input from the samples in the initial dataset, then randomly selects the features to grow the tree at each node. The trees are pruned at the end of the process when reaching a prediction. RF achieves a better performance by voting for the best result from multiple trees. In this study, the RF parameters are estimators=100 and $random_{s}tate=15$, based on exterminating with the datasets. This means that its forest consists of 100 trees and each tree is built with 10 randomly selected attributes.

**Support Vector Machines (SVMs)** can be used for both regression and classification problems [47]. The SVM algorithm draws each sample as a point in an *N*-dimensional space, where *N* is the number of features. The prediction is performed by discovering an exact hyper-plane that can define the target classes. An optimal plane is one that achieves the largest margin between the classes. SVM is best known for its scalability for large datasets, flexibility due to adjustment via a broad class of kernel functions, and its low computational complexity. In this study, SVM hyperparameters were optimized using the BayesSearchCV Scikit Optimize library [48], which performs Bayesian optimization of the model.

**Boosting Stage with LightBoost (LB):** The purpose of the training boosting algorithms is to learn the best combination of class centroids and ensemble weights that minimizes a specific objective function [44], given a set of base learners L1,L2,…Ln. The LighBoost algorithm is employed to solve this optimization problem. LB is a high-performance gradient boosting algorithm based on decision trees [42]. In general, gradient boosting algorithms sequentially add trees, one at a time, to the ensemble and fit them to correct the prediction of prior models. The proposed approach employs hyperoptimized gradient boosting (hgboost) [49], which is a python package for hyperparameter optimization for LB. Furthermore, a stochastic gradient boosting is adopted, where at each iteration, the base learners are trained on a subset of the training data samples. Data selection in each boosting iteration is adjusted to handle the class imbalance, in order to prevent our models from being biased towards the class with higher representation (MS patient samples).

## 3. Results

In this section, the results of the evaluation of the proposed approach are presented. The dataset is explained in the Multiple Sclerosis Data Description Section, and the performance metrics are outlined in the Performance Metrics Section. The experimental results are then presented. A discussion of all results obtained is given with the results of each experiment.

### 3.1. Performance Metrics

The diagnostic ability of each ML model was assessed using the metrics below.

**Accuracy** is calculated by dividing the number of correct predictions by the total number of samples to show how many times the model was correct. Accuracy is calculated using Equation (2), where:

- True Positive (TP): the number of positive instances correctly classified as positive.
- True Negative (TN): the number of negative instances correctly classified as negative.
- False Positive (FP): the number of negative instances classified as positive.
- False Negative (FN): the number of positive instances classified as negative.

(2) Accuracy=(TP+TN)/(TP+FN+TN+FP)

**Matthew’s Correlation Coefficient (MCC)** estimates the correlation coefficient between the actual and the predicted value for binary classification. The MCC uses all the values in the confusion matrix; this makes it a more reliable statistical measure, which yields a high score in all the confusion matrix values when the classifier achieves good results. The MCC value ranges from −1 to 1, where −1 signifies a completely false learning method and 1 is considered a completely correct learning method [50]. The MCC is calculated as shown in Equation (3).

(3) $$MCC=\frac{\left(TP\times TN-FP*FN\right)}{\sqrt{\left((TP+FN)\times (TN+FP)\times (TP+FP)\times (TN+FN)\right)}}$$

The **Root-Mean-Squared Error (RMSE)** represents the differences between the target samples to be diagnosed and the predicted ones. The RMSE can be estimated by taking the root of the Mean-Squared Error (MSE) for the average of the squares of the measured errors. If *x* is the target variable to be predicted, *y* is the predicted variable, and *n* is the total number of samples, then the RMSE is calculated as shown in Equation (4).

(4) $$RMSE=\sqrt{\frac{1}{n}\sum_{i=1}n{\left(x_{i}-y_{i}\right)}^{2}}$$

The **Area Under the curve (AUC)** is a graph that shows the sensitivity (true positive rate) plotted as a function of the specificity (false positive rate) using different cut-off values. The AUC is used as a measure of how well a parameter can differentiate between two classes (patient and normal cases). The AUC is a more accurate measure of the performance compared to the accuracy, especially with class imbalance datasets. A value of 0.5 for the AUC specifies random class prediction, while 0 is inverse class detection, and 1 represents perfect class detection.

**The F-score** is calculated using Equation (5). where $Precision=\frac{TP}{TP+FP}$, $Recall=\frac{TP}{TP+FN}$

(5) $$F-score=2\times \frac{Precision\times Recall}{Precision+Recall}$$

### Experiments

In this section, the evaluation of the proposed method for imbalanced data handling is presented, followed by the results of the selection of differentially expressed genes. Then, the proposed method is evaluated using the different feature sets. Finally, the Gene Ontology (GO) term, biological process, and KEGG Pathway enrichment analysis for the discovered DEGs in our study are presented.

#### 3.1.1. Evaluating the Imbalanced Data Handling Performance

To evaluate the proposed method for handling imbalanced data, five datasets were selected from the KEEL imbalanced datasets. Table 2 shows the details of these datasets. The datasets were selected based on the difference in the Imbalance Ratio (IR) between the classes. The IR represents the ratio between the number of samples among the class, where if IR = 2, it means one of the classes has a number of samples that is twice the other class. Our proposed approach was compared with state-of-the-art methods, as well as with one of the most recent ensemble methods for class imbalance problem introduced by H. Guo et al. [51]. Table 3, Table 4, Table 5, Table 6 and Table 7 show the results of the proposed technique and other techniques on the same data. Among all datasets, the proposed approach recorded the highest accuracy and AUC, while it achieved a slightly lower RMSE for some of the datasets. For classification purposes, the proposed approach is considered to outperform existing methods regrading the accuracy. As shown in the tables, our proposed approach performs well compared to the others, achieving a 95%, 96%, 94%, 95%, and 9% accuracy, with an IR of 1.87, 3.25, 5.14, 8.60, and 9.28, respectively. Even with a very high imbalance ratio, where the majority class samples are nine-times the minority class, the proposed approach achieves high accuracy and a low error rate, 92% and 0.051, for the accuracy and RMSE, respectively. The proposed approach proves its stability with a high IR as its performance metrics did not deteriorate as in other methods, such as SVM and KNN, where their performance deceased significantly with the the increase in the IR value.

#### 3.1.2. Selection of Differentially Expressed Genes

**Figure 3:**
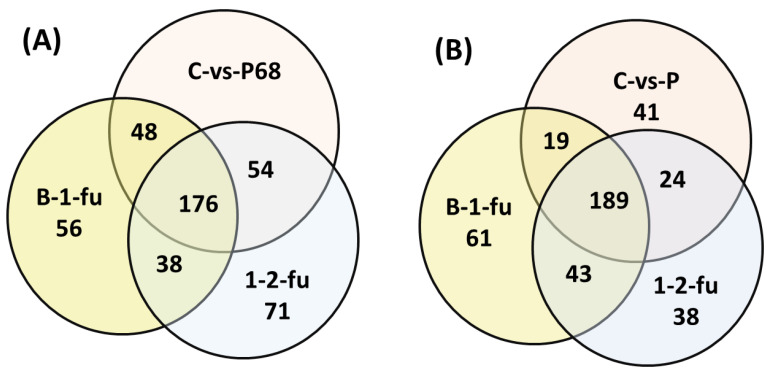
Venn diagram for Differentially Expressed Genes (DEGs) of MS for three stages; Control vs. Patient (C-vs-P), Baseline to the first-year follow-up (B-1-fu), and first-year follow-up to second-year follow-up (1-2-fu). (**A**) shows the Venn diagram of up-regulated and (**B**) shows the Venn diagram of down-regulated genes in the three MS stages.

The microarray dataset used contains 816 samples of MS patients and 126 control ones. Following the raw data preprocessing steps, the differential gene expression analysis was filtered using the FDR adjusted with *p* < 0.005. Based on the analysis of the DEGs in three different MS stages, baseline (first time of diagnosis), the first-year follow-up, and the second-year follow-up, for up-regulated and down-regulated genes, the DEGs of each MS stage were identified. The Venn diagram for the numbers of DEGs of the three stages is presented in Figure 3. As shown in the diagram, 365 genes were found to be differentially expressed in the three stages, among which 176 were up-regulated and 189 were down-regulated. These DEGs were used as the selected genes set, and their expression profiles were fed to the the proposed approach for training and testing the classifier.

#### 3.1.3. Evaluation of the Proposed MS Detection Approach

The proposed approach was evaluated on five different feature sets. Those sets are all features or feature sets selected using the chi-squared approach, RFE-SVC, BoostFS, as well as with deferentially expressed genes. For each set of features, the proposed ensemble approach was compared to random forest, KNN, and SVM. Table 8 shows the results obtained for all feature sets using different classifiers. The proposed approach recorded the highest accuracy, specifically 89.61%, 89.5%, 90.90%, 92.81%, and 91.8%, for all features, the chi-squared feature set, the RFE-SVC feature set, BoostFS, and with DEGs, respectively. Regarding the classification accuracy, the proposed approach increased the accuracy of existing methods by up to 9% among all feature sets. Moreover, the MCC of the proposed approach significantly outperformed existing methods, with an increase of up to 11%. The MCC achieved was 89% and 88%, for all features and the chi-squared feature set, while it achieved 91% for the RFE-SVC feature set, BoostFS, and with DEGs, respectively. The measures of the MCC indicate high agreement between the predicted and observed values in the proposed method. The RMSE achieved was also lower in most of the cases for the proposed approach, and the high f1-score achieved by the proposed method indicates better performance in terms of precision and recall.

**Figure 4:**
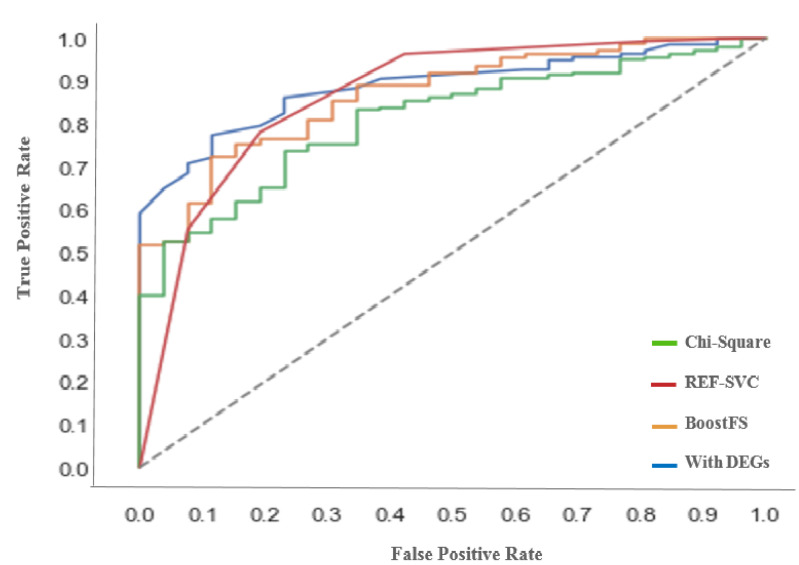
ROC curve for the proposed approach with different feature algorithms.

The Area Under the Curve (AUC) was calculated based on the ROC curve for each model to describe the quality of the work, which provides more accurate visual interpretation for the prediction performance. Figure 4 shows the ROC curve for our proposed approach with different feature selection algorithms. The curves show better performance for the proposed approach over BoostFS and DEGs. This indicates the ability of the proposed approach to distinguish between positive and negative cases correctly.

#### 3.1.4. Evaluating the Feature Selection Algorithms

**Figure 5:**
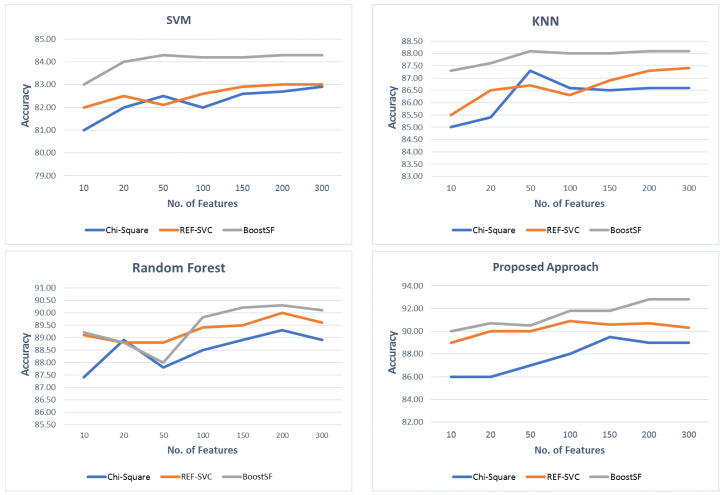
Comparison of the feature selection algorithms with different numbers of features.

This section presents comparative experiments aiming to evaluate the feature selection algorithms used in this study. The accuracy and AUC were calculated to test the algorithm for the best features set selection on the final results reported in Table 8. A set of group experiments was conducted on each algorithm with different cut-offs for selecting the number of features, starting from 5 to 1000 with the step size equal to five. The selection of five as the number of features to select first, as well as the step size, was because five is relatively small and is likely to have an inauspicious effect on the feature selection quality. The results for these experiments are reported in Figure 5. As revealed by the figure, most of the algorithms’ performance stabilized when the number of features was around 100 genes, while some algorithms’ performance deceased when the number of genes selected increased, such as RFE-SVC, where the accuracy of the classifier reduced as the number of selected features became more than 150. This is expected from a wrapper-based FS algorithm, which suffers from computational complexity, as it uses recursive feature elimination in combination with a support vector clustering (SVC) algorithm, to select important features based on the minimal error of the SVC.

#### 3.1.5. Gene Ontology and Pathway Enrichment Analysis

**Figure 6:**
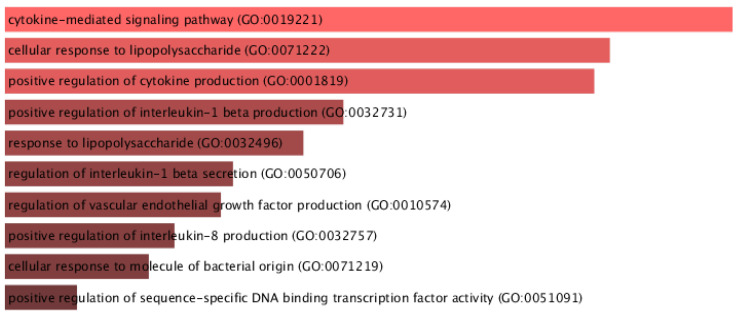
Enriched Gene Ontology terms in the identified DEGs.

The identified DEGs were analyzed by their enriched O term, biological functions, and pathways using EnrichR R package version 2.0 [34], where GO terms and biological function were selected from 2019 databases and KEGG Pathways from 2018 databases. Here, GO terms, functional processes, and KEGG Pathways are considered significant if their adjusted p-value is less than 0.05. EnrichR was used to to plot the bar diagram to show the most enriched GO terms, as shown in Figure 6, and biological processes and pathway analysis figures are shown in Supplementary Figure S1 and Figure S2, respectively.

Supplementary Table S1 (DEGs.xlsx) shows the differentially expressed genes between multiple sclerosis patients and normal samples and up- and down-regulation, with adjusted *p*-values < 0.05.

Supplementary Table S2 (KEGG_human_table.xlsx) presents the KEGG Pathways’ enrichment analysis for the DEGs in Supplementary Table S1, which revealed that the identified DEGs are closely related to NOD-like receptor signaling and Salmonella infection pathways with *p*-values < 0.05).

Supplementary Figure S1 (KEGG_Human_bar_graph.png) shows the top enriched pathways in a bar graph.

Gene Ontology (GO) enrichment analysis for the identified DEGs in MS patient samples versus normal involved protein cytokine-mediated signaling and cellular response to lipopolysaccharide processes is shown in Supplementary Table S3 (GO_Biological_Process_table.xlsx).

Supplementary Table S4 (GO_Molecular_Function_table.xlsx) presents the GO functional analysis for the identified DEGs, which proves that these DEGs are related to MAP kinase phosphatase activity and kinase tyrosine biological function with *p*-values < 0.05).

Supplementary Figure S2 (GO_Molecular_Function_bar_graph.png) shows the most enriched biological function in a bar graph.

## 4. Conclusions

This paper presented an ensemble approach using voting and boosting techniques for the prediction of multiple sclerosis patients using gene expression profiles. Two base learners, namely random forest and support vector machine, were employed with the voting and boosting techniques. Moreover, over-sampling was used to handle the class imbalance problem in the gene expression data. The proposed method for the class imbalance problem was evaluated on five KEEL imbalanced datasets, and the results obtained for the proposed over-sampling approach showed a higher classification accuracy than existing methods.

The proposed classification approach for MS was tested with different feature sets, namely all features, differentially expressed genes set, and reduced feature subsets using three feature selection algorithms: Chi-squared, recursive feature elimination with support vector regression, and Extreme Gradient Boosting (XGBoost) for feature selection (BoostFS), as well as DEGs. The proposed method was compared to existing classifiers, namely random forest, KNN, and SVM. Experimental results showed that the proposed method achieved the highest accuracy of 92.81% and 91.8% with BoostFS and DEGs, respectively, in the diagnosis of MS. Hence, the proposed method outperforms the classification accuracy, MCC, AUC, and f1-score of all existing techniques.

To the best of our knowledge, not much work in the literature has been directed towards the diagnosis of MS, especially using gene expression. Yet, the proposed approach outperforms the prediction accuracy reported in [14,15,16,17], where they reported an accuracy ranging from 68% to 95%, and not all methods concentrated on diagnosis, but some on the correlation between different variables. The proposed approach reported the highest accuracy of 92.81%, using a combination of DEGs and genes selected from recent wrapper selection methods, such as XGBoost, for a more accurate MS diagnosis. Gene expression profiles pose a challenge for ML methods, as their high-dimensional data could lead to ML algorithm over-fitting. Furthermore, most gene expression profiles’ data suffer from a class imbalance, which adds more obstacles to the learning algorithm. In this paper, we employed an over-sampling technique to solve the imbalance problem. This study could be further expanded by proposing a novel over-sampling method and experimenting on more base classifiers for our ensemble approach. Furthermore, another direction could involve deeply mining the expression data of multiple sclerosis.

## Figures and Tables

**Table 1:** MS datasets’ description.

Datasets	Classes	Features (Genes)	Patients Samples	Control Samples
**GSE41850**	Two classes: control or patient	18,722	691	126
**GSE13732**	Control or patient	21,653	85	28
**GSE24427**	Only patient samples	22,653	250	–
**GSE19285**	Only patient samples	21,147	144	–

**Table 2:** Benchmark datasets: the KEEL imbalanced datasets.

Dataset	Imbalance Ratio	No. of Samples	No. of Features
Pima	1.87	768	8
vehicle0	3.25	846	18
new-thyroid2	5.14	215	5
ecoli3	8.60	336	7
ecoli-0	9.28	257	7

**Table 3:** Evaluation results for the proposed algorithm to handle the imbalanced class problem—random forest.

Random Forest			
**Dataset**	**ACC**	**RMSE**	**AUC**
Pima	95%	0.069	0.94
vehicle0	94%	0.068	0.91
new-thyroid2	92%	0.047	0.89
ecoli3	90%	0.063	0.88
ecoli-0	91%	0.067	0.90

**Table 4:** Evaluation Results for the proposed algorithm to handle the imbalanced class problem—SVM.

SVM			
**Dataset**	**ACC**	**RMSE**	**AUC**
Pima	93%	0.051	0.94
vehicle0	95%	0.056	0.92
new-thyroid2	94%	0.041	0.95
ecoli3	89%	0.053	0.88
ecoli-0	90%	0.076	0.89

**Table 5:** Evaluation results for the proposed algorithm to handle the imbalanced class problem—KNN.

KNN			
**Dataset**	**ACC**	**RMSE**	**AUC**
Pima	94%	0.092	0.85
vehicle0	89%	0.071	0.87
new-thyroid2	90%	0.077	0.84
ecoli3	90%	0.079	0.89
ecoli-0	90%	0.072	0.90

**Table 6:** Evaluation results for the proposed algorithm to handle the imbalanced class problem—Guo et al.

Guo et al [51]			
**Dataset**	**ACC**	**RMSE**	**AUC**
Pima	95%	0.081	0.91
vehicle0	92%	0.069	0.89
new-thyroid2	91%	0.064	0.88
ecoli3	92%	0.090	0.91
ecoli-0	87%	0.097	0.84

**Table 7:** Evaluation results for the proposed algorithm to handle the imbalanced class problem—proposed method.

Proposed Method			
**Dataset**	**ACC**	**RMSE**	**AUC**
Pima	95%	0.029	0.94
vehicle0	96%	0.046	0.95
new-thyroid2	94%	0.065	0.95
ecoli3	95%	0.048	0.94
ecoli-0	92%	0.051	0.91

**Table 8:** Proposed MS detection approach evaluation.

Classification Algorithm	Feature Selection	Accuracy	MCC	RMSE	F1 Score
**SVM**	All Features	82.41%	82%	0.058	0.79
	Chi-Squared	82.50%	83%	0.064	0.81
	RFE-SVC	83.30%	84%	0.065	0.85
	BoostFS	84.81%	85%	0.061	0.86
	With DEGs	86.81%	87%	0.023	0.86
**KNN**	All Features	84.61%	84%	0.075	0.84
	Chi-Squared	86.50%	85%	0.056	0.86
	RFE-SVC	87.53%	87%	0.099	0.87
	BoostFS	88.81%	89%	0.091	0.89
	With DEGs	89.50%	86%	0.089	0.88
**Random Forest**	All Features	84.61%	85%	0.084	0.84
	Chi-Squared	88.50%	88%	0.056	0.88
	RFE-SVC	89.50%	90%	0.072	0.89
	BoostFS	90.81%	91%	0.063	0.90
	With DEGs	90.17%	89%	0.043	0.89
**Proposed Ensemble Method**	All Features	89.61%	89%	0.081	0.88
	Chi-Squared	89.50%	88%	0.043	0.89
	RFE-SVC	90.90%	91%	0.079	0.91
	BoostFS	92.81%	93%	0.067	0.93
	With DEGs	93.54%	94%	0.059	0.93

## Data Availability

The dataset is open access and is available through The GEO database (https://www.ncbi.nlm.nih.gov/geo (accessed on 26 April 2022)).

## Supplementary Materials

The following Supporting Information can be downloaded at: https://www.mdpi.com/article/10.3390/diagnostics12071771/s1.
