# Supplementary figures and images for: An Enhanced Multiple Sclerosis Disease Diagnosis via an Ensemble Approach
Source: Diagnostics (Basel). 2022 Jul 21;12(7):1771. doi: 10.3390/diagnostics12071771 (PMC9316893; doi:10.3390/diagnostics12071771)

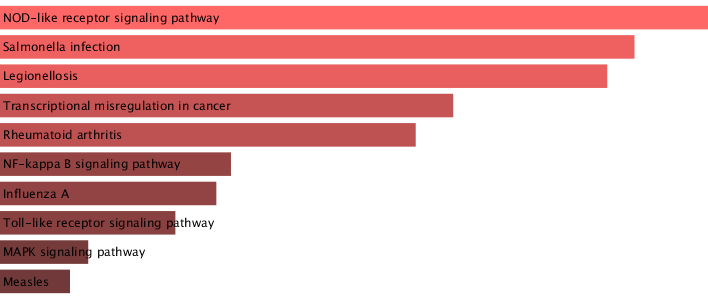

Supplement: Supplementary file 1 [file diagnostics-12-01771-s001.zip › Figure S1.png]

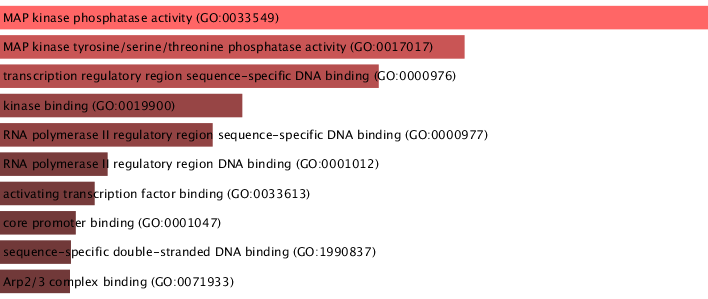

Supplement: Supplementary file 1 [file diagnostics-12-01771-s001.zip › Figure S2.png]

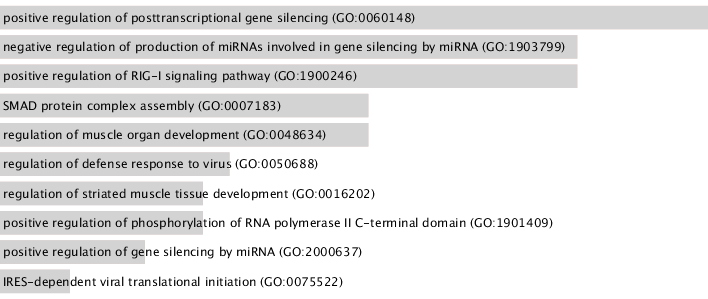

Supplement: Supplementary file 1 [file diagnostics-12-01771-s001.zip › Figure S3.png]
